# Frequency of Aneuploidy Related to Age in Porcine Oocytes

**DOI:** 10.1371/journal.pone.0018892

**Published:** 2011-04-27

**Authors:** Miroslav Hornak, Michal Jeseta, Petra Musilova, Antonin Pavlok, Michal Kubelka, Jan Motlik, Jiri Rubes, Martin Anger

**Affiliations:** 1 Institute of Animal Physiology and Genetics, Libechov, Czech Republic; 2 Veterinary Research Institute, Brno, Czech Republic; University of Texas-Houston Medical School, United States of America

## Abstract

It is generally accepted that mammalian oocytes are frequently suffering from chromosome segregation errors during meiosis I, which have severe consequences, including pregnancy loss, developmental disorders and mental retardation. In a search for physiologically more relevant model than rodent oocytes to study this phenomenon, we have employed comparative genomic hybridization (CGH), combined with whole genome amplification (WGA), to study the frequency of aneuploidy in porcine oocytes, including rare cells obtained from aged animals. Using this method, we were able to analyze segregation pattern of each individual chromosome during meiosis I. In contrast to the previous reports where conventional methods, such as chromosome spreads or FISH, were used to estimate frequency of aneuploidy, our results presented here show, that the frequency of this phenomenon was overestimated in porcine oocytes. Surprisingly, despite the results from human and mouse showing an increase in the frequency of aneuploidy with advanced maternal age, our results obtained by the most accurate method currently available for scoring the aneuploidy in oocytes indicated no increase in the frequency of aneuploidy even in oocytes from animals, whose age was close to the life expectancy of the breed.

## Introduction

Development of human embryo is affected by a high frequency of aneuploidy, which has severe consequences, namely pregnancy loss, incidence of abortions, developmental disorders and mental retardation [1]. Search for the potential source of these defects showed, that the female gametes are more susceptible to the accumulation of chromosome segregation errors during meiosis I division, and therefore the egg is the major contributor to the embryo aneuploidy [2]–[4].

Data obtained from other mammals show that their oocytes are also frequently affected by aneuploidy. In mouse, where this phenomenon was extensively studied, the frequency of aneuploidy at metaphase II, estimated by chromosome spreads or by counting CREST signals after monastrol treatment in intact oocytes, varies from 3% to 8% [5]–[7]. In cattle, the differences between individual reports are even greater, with estimated aneuploidy rate in MII oocytes varying from 7.1% up to 30%, using either chromosome spreads and counting hyperhaploid oocytes [8] or counting chromosomes X and 5 detected by FISH [9]. Analysis of porcine oocytes based on the hyperhaploid chromosome spreads showed aneuploidy in 4.9% of cells [10] or 11.9% cells [11], whereas counting chromosomes 1 and 10 using FISH led to the estimated aneuploidy ranging from 27% up to 57% of oocytes [12], [13].

The development of mammalian female gametes is characterized by a relatively long interruption, lasting from the formation of oocytes during early embryonic development until their recruitment to complete meiosis after puberty. The length of this period, during which are the oocytes arrested in the prophase of the first meiotic division, varies dramatically between species and can last from months to decades. It seems that the frequency of aneuploidy increases significantly with declining reproduction, which places increased maternal age within the potential risk factors of developing embryo suffering from aneuploidy. Analysis of the correlation between age of human female donors and oocyte aneuploidy showed that in young women relatively small fraction, around 3–10% of oocytes, are aneuploid, whereas in women in their forties and later, the frequency of aneuploidy exceeds 50% [14]–[16]. To our knowledge, the only non-human mammalian species, in which the correlation between maternal ageing and oocyte aneuploidy was systematically studied, was mouse. In this species, the low overall initial aneuploidy 3–8% in animals around age of 8–10 weeks increases to 12% at the age of 32 to 35 weeks and even further increases to 25% at the age of 70 weeks, these results were obtained by counting chromosomes on chromosome spreads [6], [7]. The high frequency of aneuploidy in animals advanced in age was confirmed also by a different method, namely disruption of the metaphase II spindle by monastrol and counting kinetochores on chromosomes stained by DAPI and CREST [5]. Using this method authors showed that the incidence of aneuploidy in oocytes from animals at the age of 16–19 months (64–76 weeks) is as high as 35%.

Due to the differences between various techniques used for scoring aneuploidy in oocytes, the reported frequencies are sometimes highly heterogeneous [17]. The objective of our study was to obtain the most accurate picture of the incidence of aneuploidy in porcine oocytes, since the previously published data are rather inhomogeneous. We were also keen to know, whether in this species the frequency of aneuploidy in oocytes increases in correlation to the maternal age. Our aim was to obtain physiologically more relevant model system to study maternal age-related aneuploidy in oocytes, because the meiosis in porcine oocytes resembles much more the situation in human than in rodent oocytes, generally used for such studies. For scoring the aneuploidy in oocytes in our study, we employed comparative genomic hybridization (CGH), which proved to be more accurate for scoring chromosomal abnormalities than FISH [18].

Our data presented here show that the incidence of aneuploidy in porcine oocytes is lower than the most frequent estimations reported previously. Surprisingly, comparison of the frequency of aneuploidy in groups of animals of age more than 6 years apart showed that this species does not suffer from age-related increase of aneuploidy in oocytes, known in human and mouse.

## Results

### In vitro maturation of oocytes from various age categories

Oocytes isolated from two groups of miniature pigs and a group of Landrace and Czech Large White crossbreed (LxCLW), were matured in vitro. First group of miniature pigs consisted of three young animals at average age around 15 months. Animals in the second group were significantly older, with average age around 92 months. Oocytes in the third group, obtained from the LxCLW, were in average 69 months old at the time of oocyte isolation. GV stage oocytes, with intact cumulus (COCs), were isolated after prior PMSG stimulation. Within each group, similar number of COCs per animal was obtained, 14.67 from young miniature pigs, 19.34 from aged miniature pigs and 27 from the group of aged LxCLW (Table 1). After 44 hours of maturation in vitro, 77.3, 82.8 and 87.7% of oocytes isolated from the young miniature pigs, aged miniature pigs and LxCLW extruded the first polar body (PB) (Table 1). The rate of polar body extrusion is comparable to the data from other groups indicating that our culture conditions were supporting oocyte maturation in vitro [13]. For subsequent analysis, the polar bodies were detached from oocytes and frozen separately.

**Table 1:** In vitro maturation of porcine oocytes isolated from various experimental groups.

Experimental group	Animals per group	Average age (months ± SD)	Number of isolated oocytes	Maturation rate number/% of PBs
Miniature pigs young	3	15.33±4.16	44	34/77.3
Miniature pigs old	3	92.00±21.66	58	48/82.8
LxCLW old	3	69.00±1.73	81	71/87.7

The number of animals per group, their average age, total number of isolated oocytes and the maturation rate scored after 44 hours of in vitro culture represented by absolute number of oocytes with polar bodies and frequency of polar body extrusion per group is indicated for each group. LxCLW stands for crossbred of Landrace and Czech Large White pigs.

### Comparative genomic hybridization (CGH) of the entire chromosome content of individual oocytes

**Figure 1:**
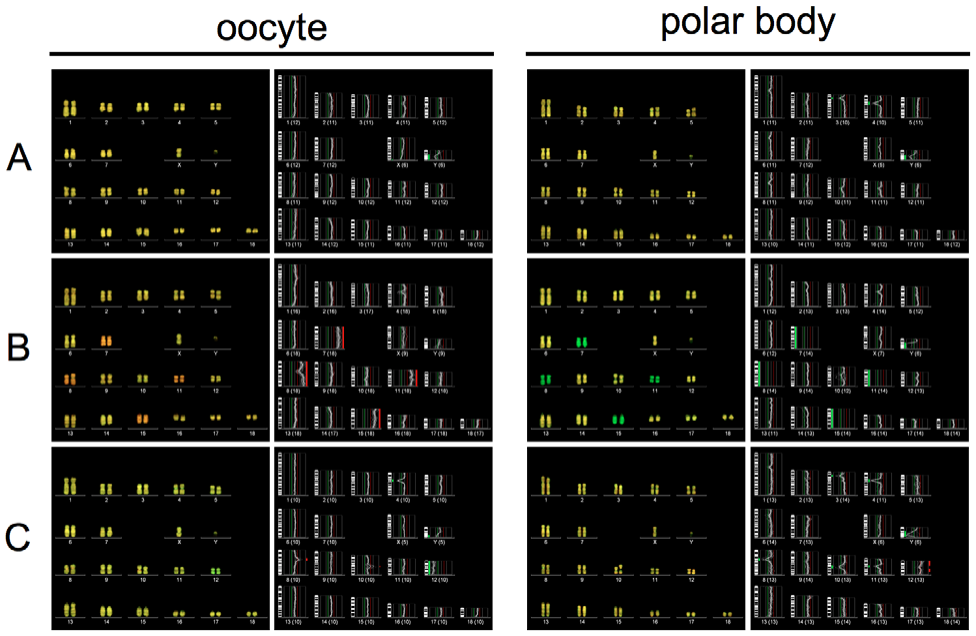
Detection of chromosome segregation errors by comparative genomic hybridization (CGH). Panel A: CGH analysis of euploid oocyte (left panel) and the corresponding polar body (right panel). Panel B: CGH analysis of oocyte (left panel) and the corresponding polar body (right panel) with non-disjunction of chromosomes 7, 8, 11 and 15. Panel C: CGH analysis of oocyte (left panel) and the corresponding polar body (right panel) with premature segregation of sister chromatids of chromosome 12.

CGH method, when DNA template comes from a single cell, represents a technical challenge, since the amount of template DNA in the reaction is drastically reduced. To solve this problem, we used amplification of the whole genome using commercially available PicoPlex™ WGA kit (Rubicon Genomics). This technology was succesfully validated for aneuploidy detection in human blastomeres and polar bodies [19], [20]. Our preliminary results showed that CGH from a single porcine oocyte is feasible and reproducible. We have observed a strong correlation between aneuploidy detected in the oocyte and the corresponding polar body (data not shown). In Figure 1, we summarized representative results of CGH analysis. Oocyte with normal chromosomal set and the corresponding polar body is shown on panel A. The yellow mixed hybridization signal, consisting of green (Green 496-dUTP) signal from control cumulus cell and red (Cyanine 3-dUTP) signal from the oocyte or the polar body, is uniformly covering each chromosome (ratio close to 1∶1). A result of a segregation error, when the whole bivalents were not disjoined during meiosis I, is illustrated on panel B. Chromosomes 7, 8, 11 and 15 are predominantly labeled in red in the oocyte and entirely green in the polar body (see corresponding ratio profile), indicating that those chromosomes remained in the oocyte during the first meiotic division. Panel C illustrates situation, when sister chromatids separated prematurely during meiosis I. The red signal of chromosome 12 is not completely missing, however, the green signal of competing control DNA is more apparent (signal ratio shifted to green, see ratio profile). Importantly, the color of the signal from the same chromosome in the corresponding PB represents the dominant signal from tested cell (signal ratio shifted to red, see ratio profile). Presented results demonstrate, that when using our assay we are able to detect the most frequent sources of aneuploidy in mammalian oocytes – failure to disjoin the whole bivalent or premature segregation of sister chromatids.

### Frequency of aneuploidy related to the donor age

The chromosome content was analyzed in 141 oocytes. From the final score, three cells from the group of aged LxCLW were excluded, because of the failure of the sample preparation (more than half of the chromosomes missing in the oocyte with euploid PBs), and the result of CGH analysis of 138 oocytes are summarized in Table 2 and 3. Altogether, we have identified 14 oocytes with incorrect chromosome content. In 9 cases, the aneuploidy was confirmed by CGH of the corresponding polar body. In two cases, amplification of DNA from the polar body failed and in three cases the profile of the polar body did not confirm the aneuploidy found in the oocyte. In the cases, where we were able to confirm the aneuploidy by analyzing the chromosome content in PBs, the affected chromosomes in the oocyte were accurately reflected in the corresponding PB. Both major causes of oocyte aneuploidy were present, 4 cells showed nondisjoined chromosomes and 10 cells suffered from premature segregation of sister chromatids (PSSC) (Table 3). When we divided the oocytes according to the age of donor animals, the frequency of aneuploidy was similar in oocytes from young miniature pigs, (12.1%), in oocytes from aged miniature pigs (8.7%) and in oocytes obtained from aged farm animals (LxCLW – 10.2%) (Table 2).

**Table 2:** The frequency of aneuploidy in oocytes from young donors versus oocytes from aged donors.

Experimental group	Total number of oocytes analyzed by CGH	Total number of oocytes with aneuploidy	Frequency of aneuploidy (%)
Miniature pigs young	33	4	12.1
Miniature pigs old	46	4	8.7
LxCLW old	59	6	10.2

Table summarizing the frequency of aneuploidy in oocytes isolated from various age categories. The total number of oocytes analyzed by comparative genomic hybridization (CGH) together with the number of oocytes with incorrect chromosomal counts and their frequency per group is indicated. LxCLW stands for crossbred of Landrace and Czech Large White pigs.

**Table 3:** The analysis of the chromosomal segregation errors of porcine oocytes matured in vitro.

Experimental group	Chromosomes contributing to the aneuploidy - oocytes		Chromosomes contributing to the aneuploidy - PBs
	nondisjunction	PSSC	
Miniature pigsyoung	−2, −8, −15		Euploid
	+13, +14, +15		Unsuccessful amplification
		+15	−15
		−9	+9
Miniature pigsold	+7,+8,+11,+15		−7, −8, −11, −15
	+5		−5
		+12	−12
		−12	+12
LxCLWold		−6	Euploid
		−8, −13	Unsuccessful amplification
		−13	Euploid
		−1	+1
		−2	+2
		+X	−X

Oocytes with incorrect number of chromosomes are shown for each group. The origin of aneuploidy (chromosome non-disjunction versus premature segregation of sister chromatids - PSSC), chromosomes contributing to aneuploidy and the result of analysis of the corresponding polar body are shown for each cell. LxCLW stands for crossbred of Landrace and Czech Large White pigs.

## Discussion

We show for the first time the CGH analysis of the whole chromosome content of a single female germ cell from non-human species. According to our results the frequency of aneuploidy in 138 analyzed porcine oocytes is 10.1%. These findings correspond to the large karyotyping survey of 1,397 human metaphase II oocytes, in which the frequency of aneuploidy was 10.8% [16]. Surprisingly, we were not able to detect increased frequency of aneuploidy in oocytes isolated from aged animals. In the group of oocytes isolated from young animals, 12.1% were aneuploid compared to aneuploidy ranging from 8.7% to 10.2% scored in oocytes isolated from two different breeds of aged animals. The low level of aneuploidy in oocytes from aged animals was unexpected and surprising; since in human and in mouse it seems that the level of aneuploidy increases with maternal age [1], [6]. The simplest explanation could be that the animals, which we used for our analyses, were still relatively young and therefore the rate of aneuploidy was still low. Although we cannot exclude this possibility completely, it is very unlikely. Firstly, the estimated lifespan of miniature pig breed is between 10–15 years [21] and in our group of aged animals of this breed we have individuals 6, 8 and 9.5 years old, which should be close to the end of the expected lifespan. Secondly, we do not see any increase of the frequency of aneuploidy between young and aged animals, although in our study we are comparing animals separated by, on average, 6.39 years (average age in the group of young miniature pigs was 15.33 ± 4.16 months and in the group of old miniature pigs 92.00±24.66 months) with the biggest difference being 8.5 years (data not shown). It is reasonable to assume that such substantial age difference would somehow influence the frequency of aneuploidy, even if our animals were not at the end of their lifespan yet. We can also exclude a possibility that the data are reflecting only conditions within a particular breed of animals, since we are comparing two breeds with different genetic background - miniature pigs and a crossbreed of Landrace and Czech Large White – and the results in both groups are similar.

The CGH seems to be very reliable tool compared to the highly variable results produced by traditional methods used for scoring aneuploidy in oocytes [17]. Although this method was successfully used before to analyze the level of aneuploidy in porcine embryos [22], for the analysis of a single oocyte or polar body presented here, it was necessary to modify several steps, including whole genomic amplification (WGA) procedure. The major advantage of CGH over traditional methods is that the chromosomes are not extracted before cell lysis and WGA, therefore the problem of loosing the chromosomes during procedure, a major drawback of chromosome spreading techniques and SKY karyotyping, is eliminated here. Also, in contrast to the FISH method, when only few individual chromosomes could be detected, here we are able to analyze the entire chromosome set. This for example means that although we have analyzed chromosome content of merely 138 cells in our study, in fact we have analyzed precisely the segregation pattern of 2622 chromosomes. Additionally, most of the cases of aneuploidy detected in oocytes were confirmed also by analysis of the DNA in the corresponding polar body.

Chromosomes have various shapes, which is probably the reason why contribution of individual chromosomes to the aneuploidy is not random [23]. Because of the relatively small set of oocytes in this study, we are not able to provide statistical results of a contribution of different chromosomes into aneuploidy. However, we have noticed that chromosome 15 was involved more frequently in various cases in which aneuploidy was detected. In three cases the chromosome 15 was not disjoined and in one case sister chromatids of chromosome 15 segregated prematurely in meiosis I.

From our study we can conclude that the frequency of aneuploidy in porcine oocytes, measured by CGH, is lower than it was previously published. We were also unable to detect an increase in the frequency of aneuploidy in oocytes isolated from old animals, although it is important to emphasize the importance of future studies aiming to analyze this situation on larger number of animals and also different breeds. This might reflect some fundamental differences between human, mouse and pig folliculogenesis and meiosis resulting in low frequency of aneuploidy in aged pigs. Although human and mouse oocytes both show increased frequency of aneuploidy related to donor age, whether the etiology of aneuploidy in both cases is the same, is not so clear [24]. The low level of aneuploidy detected in porcine oocytes might be also a result of the constant pressure and selection focused on reproduction and fertility in farm animals, which could eventually eliminate individuals with potential problems. The maternal age-related aneuploidy in oocytes is an important issue in human reproductive medicine, although our results presented here indicate, that some mammalian species might not be affected. In order to find out how widely is this phenomenon spread among other mammals and also to identify species which would complement the rodent model system for studying this problem, we need to analyze carefully the incidence of this phenomenon in other mammals with longer lifespan using methods allowing for analysis of the whole set of chromosomes, such as CGH.

## Materials and Methods

All animal work was conducted according to Act No 246/1992 Coll., on the protection of animals against cruelty under supervision of Central Commission for Animal Welfare, approval ID 018/2010.

### Isolation of cumulus oocyte complexes (COCs) and in vitro maturation

A total of 6 of laboratory miniature pig cycling gilts and 3 Landrace x Czech Large White animals were used as oocyte donors. Their estrus cycle was synchronized by intramuscular injection of 750 IU and 1000 IU for miniature pigs and large animals respectively PMSG on the 15th day of cycle. Ovaries were collected 62–64 hr after PMSG stimulation. COCs were isolated from large preovulatory follicles and washed three times in M-199 (Sevac, Prague, Czech Republic), buffered with 6.25 mM HEPES and 26 mM sodium bicarbonate and supplemented with 0.91 mM sodium pyruvate, 1.62 mM calcium lactate, and antibiotics. COCs surrounded by compact multilayered cumulus were cultured in the above mentioned basic culture medium supplemented with 10% inactivated estrous cow serum (prepared in our laboratory) and 5 IU mL gonadotropins PG600 (Intervet, International B.V. Boxmeer, Holland) in 0.5 ml volume per one well, in 4-well culture dishes (Nunclon, Roskilde, Denmark) for 44 hours at 38.5°C, 5% CO_2_ and 7.5% O_2_.

### Preparation of oocytes and polar bodies (PBs) for analysis

Before harvesting, the COCs were treated shortly by 0.1% (w/v) hyaluronidase (Sigma Aldrich) in culture media to remove cumulus cells. Denuded oocytes were washed five times in 10 µl droplets of PBS with 0.4% bovine serum albumin (BSA). Only oocytes with visible PB were used for analysis. Zona pellucida was removed using 0.1% (w/v) Pronase (Sigma Aldrich). During this step, PBs were separated from oocytes using very narrow-bore glass pipette and after washing in three 10 µl drops Tris-HCl (10 mM, pH 8.5), both were transferred into individual PCR tubes containing 2 µl Tris-HCl and stored at −80°C before analysis. Precautions against DNA contamination were taken when handling and sampling oocytes and corresponding PBs.

### Whole genome amplification and comparative genomic hybridization

Oocytes or polar bodies (PBs) underwent lysis and whole genome amplification using PicoPlex™ WGA kit (Rubicon Genomics) according to the manufacturer's instructions. Successful amplification of the samples was checked by agarose gel electrophoresis. A blank sample was included as a negative control with every amplification batch. Amplified DNA from oocytes and PBs was labelled by BioPrime® Array CGH Genomic labeling System (Invitrogen) using Cyanine 3-dUTP (Enzo Life Sciences) fluorescent dye. Reference DNA, prepared from single diploid cumulus cell, was also amplified and labeled using Green 496-dUTP (Enzo Life Sciences) fluorescent dye. Subsequently, comparative genomic hybridization was performed according to previously published protocol [22]. CGH analysis criteria were as follows: red signal: green signal ratio of >1.25∶1 was indicative of chromosomal material gain, while ratio of <0.75∶1 indicated loss. These criteria allow us to detect the non-disjunction of the whole bivalents as well as the pre-division of sister chromatids. The procedure of analysis of digital images was described in [22].
